# The Pharmacological Mechanisms Underlying the Protective Effect of Ginsenoside Rg3 against Heart Failure

**DOI:** 10.1155/2024/3373410

**Published:** 2024-07-30

**Authors:** Yanan Jia, Miao Gong, Zunping Ke

**Affiliations:** Department of Geriatrics Shanghai Fifth People's Hospital Fudan University, Shanghai, China

## Abstract

**Background:**

Heart failure represents the terminal stage of various cardiovascular diseases. This study aims to explore the pharmacological mechanisms underlying the protective effect of Ginsenoside Rg3 against heart failure.

**Methods:**

Potential targets of Ginsenoside Rg3 were identified using SwissTargetPrediction and the Comparative Toxicogenomics Database, while heart failure-related genes were retrieved from the Comparative Toxicogenomics Database, Therapeutic Target Database, DisGeNET, and PharmGKB. Overlapping of Ginsenoside Rg3 targets with heart failure-related genes identified drug-disease interaction genes. Gene Ontology (GO) and Kyoto Encyclopedia of Genes and Genomes (KEGG) enrichment analyses were conducted on the drug-disease interaction genes to elucidate their biological functions. A protein-protein interaction network was constructed using the drug-disease interaction genes, and the hub genes were identified by topological analysis. Additionally, we validate the expression of IL-6 and TNF by real-time PCR.

**Results:**

The intersection of Ginsenoside Rg3 targets and heart failure-related genes yielded 15 drug-disease interaction genes. Enrichment analysis highlighted the involvement of inflammation-related GO terms and KEGG pathways, such as positive regulation of interleukin-8 and -6 production, regulation of immune effector process, cytokine receptor binding, cytokine activity, adipocytokine signaling pathway, and IL-17 signaling pathway, which are implicated in the cardioprotective effect. Topological analysis revealed four hub genes: *STAT3*, *CASP3*, *TNF*, and *IL-6*. The application of Ginsenoside Rg3 significantly reversed the elevated levels of IL-6 and TNF in the isoproterenol-treated H9c2 cell line.

**Conclusions:**

Our findings suggest that the cardioprotective effect of Ginsenoside Rg3 may be mediated through its anti-inflammation properties. Further research is required to elucidate and validate the detailed cardioprotective mechanisms of Ginsenoside Rg3.

## 1. Introduction

Ginsenoside Rg3 is a prominent bioactive compound extracted from the roots of Panax ginseng [1], which has long been revered in traditional Asia herbal medicine for its wide-ranging therapeutic properties. Despite its low proportion, ginsenoside Rg3 demonstrates a pharmacological potency comparable to other well-known ginsenoside saponins, such as Rb1 and Rg1 [2–4]. This comprehensive pharmacological profile has garnered increased research interest, particularly highlighting its anti-inflammation [5], antioxidant [6], anticancer [7], and antiageing capabilities [8]. These distinct pharmacological actions have led to its use in treating various metabolic or ageing-related diseases, including cardiovascular diseases, fatty liver diseases [9], obesity [10], and diabetes [11].

Heart failure (HF) is a prevalent and multifaceted clinical syndrome characterized by the inefficiency of heart in pumping sufficient blood to meet the body's demands [12]. Recognized as the terminal stage of cardiovascular diseases, HF presents various symptoms such as shortness of breath, leg swelling, fatigue, and irregular heartbeat [12]. The ageing population has made HF a significant global public health issue, affecting an estimated 80 million people worldwide, including 16 million in China [13]. The causes of compromised cardiac function are diverse, encompassing coronary artery disease, hypertension, diabetes, and valvular heart diseases. The management of HF involves lifestyle changes, pharmacological treatment, and, for severe cases, the use of devices or surgical interventions. Despite progress in therapeutic approaches, HF continues to pose a substantial public health challenge due to its high morbidity, frequent hospital readmissions, and diminished life quality [13]. There is an ongoing need for the development of effective treatments for HF that can alleviate symptoms and extend patient survival.

The cardiovascular benefits of Ginsenoside Rg3 have gained attention in recent studies, underscoring its potential in addressing cardiovascular health [14, 15]. It has been recently reported that Rg3 could alleviate myocardial hypertrophy and fibrosis in rat models with transverse aortic constriction-induced heart failure [16]. However, the pharmacological mechanisms underlying the protective effect of Ginsenoside Rg3 against heart failure remain to be determined. Network pharmacology analysis provides a promising tool to investigate the complex interactions between drugs and diseases [17]. Diverging from the traditional pharmacological focus on single targets, network pharmacology adopts a system-based perspective, facilitating the exploration of drug interactions with multiple targets. This approach provides a comprehensive understanding of therapeutic effects and mechanisms. Recently, network pharmacology has been increasingly employed to identify new drug uses, predict side effects, and decipher the underlying mechanisms of traditional medicines [18–20]. Its scope extends beyond drug discovery, shedding light on the mechanisms underlying disease pathogenesis and informing potential therapeutic strategies.

In light of these advancements, our study employs network pharmacology analysis to examine the protective effect of Ginsenoside Rg3 against heart failure.

## 2. Methods

### 2.1. Ginsenoside Rg3 Structure Acquirement

**Figure 1:**
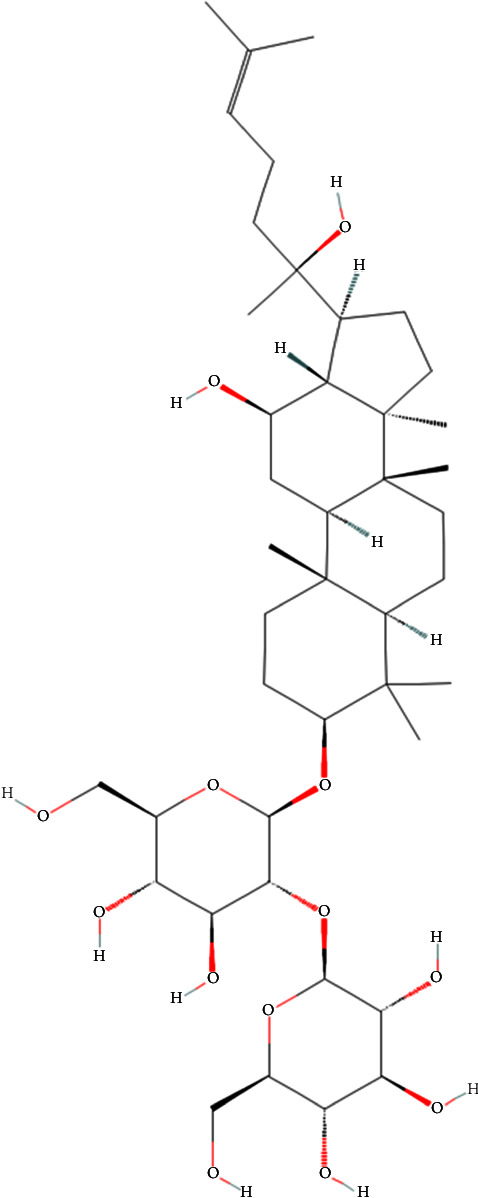
The 2D chemical structure of Ginsenoside Rg3.

PubChem, curated by the National Center for Biotechnology Information, serves as the cornerstone in the realm of chemical informatics, housing a comprehensive repository of chemical compounds, structures, and extensive associated data [21]. PubChem platform provides a powerful tool to explore molecular structures alongside their biological activities. This study utilized PubChem to investigate the molecular structure of Ginsenoside Rg3 by searching its compound name. We retrieved the 2D chemical structure and Canonical SMILES of Ginsenoside Rg3 (PubChem CID, 9918693). The Canonical SMILES was generated by OEChem 2.3.0, and the detailed SMILES is as follows: CC(=CCCC(C) (C1CCC2(C1C(CC3C2(CCC4C3(CCC(C4(C)C)OC5C(C(C(C(O5)CO)O)O)OC6C(C(C(C(O6)CO)O)O)O)C)C)O)C)O)C (Figure 1). This Canonical SMILES serves as a basis for subsequent drug target predictions.

### 2.2. Ginsenoside Rg3 Target Fishing

We inquired SwissTargetPrediction [22] and Comparative Toxicogenomics Database (CTD) [23] to identify the targets of Ginsenoside Rg3. SwissTargetPrediction, accessible at https://www.swisstargetprediction.ch, is an advanced computational platform designed to predict the most likely protein targets of small molecules [22]. By leveraging 2D and 3D similarity measures with known ligands, this tool facilitates insights into potential biological activities, off-target effects, and repurposing opportunities for compounds under investigation. SwissTargetPrediction has become a pivotal resource in both academic and pharmaceutical research for uncovering molecular mechanisms, advancing drug discovery, and developing therapeutic approaches [22]. With the species set as Homo sapiens, we input the Canonical SMILES of Ginsenoside Rg3 into the SwissTargetPrediction to predict the target predictions of Ginsenoside Rg3. The targets with low probability (<0.01) were excluded.

The CTD, accessible at https://ctdbase.org/, is a premier resource that delineates the intricate interactions among environmental chemicals, genes, and human health [23]. Founded on the rigorous manual curation of peer-reviewed literature, the CTD offers an integrated perspective on evidence-based interactions and pathways. It provides valuable insights into the mechanisms through which chemicals affect health outcomes. By detailing chemical-gene/protein interactions and their associated disease connections, the CTD serves as an essential resource to understand toxicogenomic links in human health [23]. For our study, we entered the chemical keyword “Ginsenoside Rg3” into the CTD and retrieved its target interactions.

### 2.3. Acquirement of Heart Failure-Related Genes

In addition to the CTD database [23], our research utilized the Therapeutic Target Database (TTD) [24], DisGeNET [25], and PharmGKB [26] to collect information on disease-related genes. For CTD, we identified genes associated with heart failure by entering the disease keyword of “heart failure” (MeSH ID: D006333). Moreover, the TTD Database, accessible at https://db.idrblab.net/ttd/, is a comprehensive resource that provides detailed insights into both established and exploratory therapeutic protein and nucleic acid targets [24]. This database includes data on corresponding drugs and ligands, covering approved substances as well as those under investigation. Designed to bridge gaps in drug discovery and therapeutic target research, the TTD offers a structured approach to understanding the relationships between drug development processes and target mechanisms. It encompasses targets associated with successfully marketed drugs, targets of drugs in clinical trials, and potential targets under preliminary investigation. We searched for targets related to heart failure using the “Search Drugs and Targets by Disease or ICD Identifier” function within TTD.

DisGeNET (https://www.disgenet.org/) is a leading platform dedicated to aggregating and disseminating information on gene-disease associations [25]. It integrates data from expert-curated repositories, genome-wide association studies, and scholarly literature, forming a comprehensive database of genes and variants associated with human diseases. The dataset ranges from well-established to emerging, less-explored associations. DisGeNET provides tools for analysis, visualization, and annotation, supporting researchers, clinicians, and bioinformaticians in their exploration of the genetic bases of diseases. As genomics and personalized medicine progress, DisGeNET remains a crucial resource, enhancing our understanding of disease mechanisms and aiding in the development of potential therapeutic interventions [25]. We utilized the “Summary of Gene-Disease Associations” function to identify genes related to heart failure. Only genes with a gene-disease association score higher than 0.3 from the CTD were included, ensuring the relevance and robustness of the associations.

PharmGKB (https://www.pharmgkb.org/) is a premier repository that explores the complex interactions between human genetics and drug responses [26]. Managed by a team of dedicated curators, this database systematically assembles data on pharmacogenomic genes, drug-gene interactions, and genotype-phenotype relationships. Information was recorded from a wide array of resources, including clinical and variant annotations, drug pathways, and dosing guidelines. This collection of data offers vital insights into how genetic variations affect drug efficacy, side effects, and metabolic processes. As a crucial component of personalized medicine, PharmGKB provides rigorously curated knowledge that aids in the formulation of personalized treatment plans tailored to individual genetic profiles [26]. Genes related to heart failure were identified from PharmGKB using the search term “heart failure (PharmGKB ID: PA444370).”

### 2.4. The Identification of Drug-Disease Interaction Genes

After gathering the target genes of Ginsenoside Rg3 and those associated with heart failure from various databases, we initially removed duplicate genes within each gene set. Subsequently, we identified the overlap between the Ginsenoside Rg3 target genes and heart failure-related genes. This step allowed us to isolate potential drug-disease interaction genes, which facilitates the identification of specific genes that may play a key role in the therapeutic efficacy of Ginsenoside Rg3 in treating heart failure.

### 2.5. Protein-Protein Interaction Network Construction

Protein-protein interactions (PPIs) are essential for comprehending the molecular basis of complex biological processes [27]. A detailed exploration of PPIs can elucidate the potential therapeutic mechanisms, pathways, and hub genes involved in the protective effect Ginsenoside Rg3 against heart failure. The STRING (Search Tool for the Retrieval of Interacting Genes/Proteins) platform is a comprehensive database for both known and predicted PPIs, encompassing both direct (physical) and indirect (functional) associations [28]. It is a key resource in network pharmacology owing to its comprehensive data and reliability. In this study, we utilized the STRING database to gather PPI information and construct a network based on the interactions between Ginsenoside Rg3 and heart failure.

We input candidate genes from our previous analysis into the STRING database, applying a medium confidence cut-off score of 0.4 to balance specificity and inclusiveness in PPI selection. The PPI network was then visualized using Cytoscape software (version 3.9.1) [29]. The structural and topological parameters of the PPI network were analyzed using the cytoHubba plugin within Cytoscape [30]. CytoHubba is designed to pinpoint and prioritize critical hubs within biological networks, using various topological algorithms to identify nodes of central importance to biological functions or diseases. This analysis facilitated the identification of hub genes based on the Maximal Clique Centrality algorithm within cytoHubba. The top 4 genes with the highest topological significance were identified as hub genes in the network.

### 2.6. Enrichment Analysis of the Drug-Disease Interaction Genes

To delve deeper into the biological significance of the drug-disease interaction genes associated with the effect of Ginsenoside Rg3 against heart failure, we employed the ClusterProfiler algorithm for enrichment analysis [31]. ClusterProfiler is distinguished for its ability to conduct comprehensive enrichment analysis and can effectively organize genes into biologically relevant categories [31]. We undertook both Gene Ontology (GO) [32] and Kyoto Encyclopedia of Genes and Genomes (KEGG) enrichment analyses [33]. The GO analysis illuminated the gene involvement in various biological processes, molecular functions, and cellular components. KEGG analysis offered insights into crucial pathways and molecular interactions that Ginsenoside Rg3 may influence. The *P* value was adjusted for multiple testing using the Benjamini–Hochberg procedure. Together, these combined analyses afford a thorough understanding of the gene sets, facilitating an integrated view of the molecular mechanisms.

### 2.7. Cell Culture

H9c2 rat cardiomyocyte cell line, widely used as an in vitro cardiomyocyte model, was obtained from the Chinese Academy of Medical Sciences. H9c2 cells were cultured in DMEM supplemented with 10% fetal bovine serum and were under a 37°C humid environment containing 5% CO_2_ and 95% O_2_. H9c2 cells were given isoproterenol (50 *µ*M) to induce a heart failure cell model [34]. Ginsenoside Rg3 was purchased from Shanghai Yuanye Biotechnology Co., Ltd., Shanghai, China. Ginsenoside Rg3 was added to isoproterenol-treated H9c2 cells at a concentration of 50 *μ*M [35].

### 2.8. Real-Time PCR

Total RNA was extracted from H9c2 cells and reversed within 1 h after harvest and was subjected to cDNA amplification and purification. Real-time PCR was performed using the 7500 Fast Real-Time PCR System according to the manufacturer's instructions. Each sample was analyzed in triplicate, and the data are presented as the fold change normalized to GAPDH. The primer sequences are listed in Supplement Table 1.

### 2.9. Statistical Analysis

Data were given in the mean ± standard error of the mean. One-way analysis of variance, followed by Tukey's post hoc test, was applied in multiple comparisons between the groups. GraphPad Prism 8 and R software were used for statistical analysis. *P* < 0.05 indicated a statistically significant difference.

## 3. Results

### 3.1. Candidate Drug-Disease Interaction Genes

In our investigation of the protective effects of Ginsenoside Rg3 against heart failure, we initially identified Ginsenoside Rg3 target genes and heart failure-related genes from multiple databases. Specifically, we retrieved 17 target genes of Ginsenoside Rg3 from SwissTargetPrediction and 70 genes from the CTD database.

Then, for heart failure-related genes, our search yielded 260 genes from the CTD, 77 from the TTD, 132 from DisGeNET, and 21 from PharmGKB. After eliminating duplicates, we compiled a list of 334 unique genes associated with heart failure.

Subsequently, by intersecting the Ginsenoside Rg3 target genes with the heart failure-related genes, we identified 15 genes that serve as potential drug-disease interaction candidates. These genes (listed in Table 1) are considered crucial for understanding the interaction between Ginsenoside Rg3 and heart failure mechanisms. Additionally, a comprehensive summary of these genes based on GeneCards is provided in Supplement Table 2.

### 3.2. PPI Network and Hub Genes

**Figure 2:**
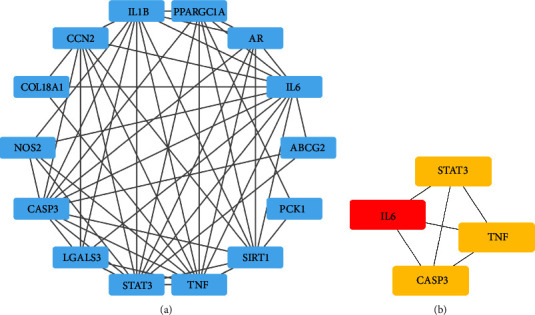
Protein-protein interaction network and hub genes. (a) The PPI network of the drug-interaction genes. (b) The top 4 hub genes identified by the Maximal Clique Centrality algorithm, including STAT3, CASP3, TNF, and IL-6.

The PPI network of the drug-disease interaction genes associated with the effect of Ginsenoside Rg3 on heart failure was constructed and is illustrated in Figure 2(a). Within this network, ATP1A1 was excluded due to its lack of interaction with other proteins, indicating its peripheral role or minimal involvement in the context of this specific drug-disease relationship. The constructed PPI network comprised 53 edges and had an average node degree of 7.07. Four hub genes were identified by the Maximal Clique Centrality algorithm, including STAT3, CASP3, TNF, and IL-6. These genes are depicted in Figure 2(b) and represent key nodes within the network, suggesting their significant roles in mediating the protective effects of Ginsenoside Rg3 against heart failure.

### 3.3. Gene Ontology Enrichment

**Figure 3:**
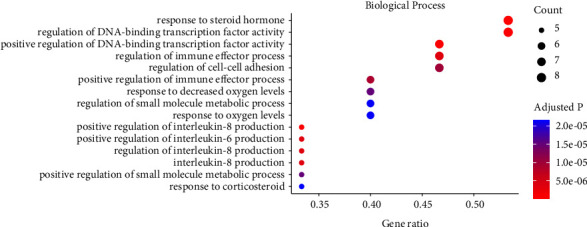
The enriched biological process terms of the Gene Ontology enrichment.

The GO enrichment analysis on the identified drug-disease interaction genes revealed biological insights categorized in three domains: biological process (BP), molecular function (MF), and cellular component (CC). The enriched BP included response to steroid hormone, positive regulation of interleukin-8 production, regulation of immune effector process, positive regulation of interleukin-6 production, regulation of interleukin-8 production, and positive regulation of immune effector process (Figure 3).

**Figure 4:**
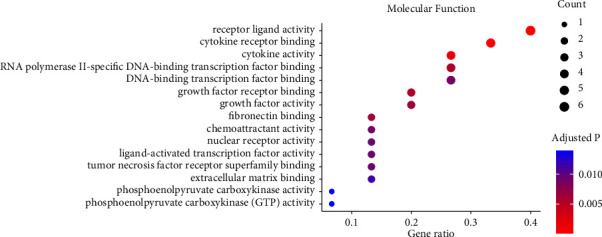
The enriched molecular function terms of the Gene Ontology enrichment.

The enriched MF terms were receptor ligand activity, cytokine receptor binding, cytokine activity, growth factor activity, and fibronectin binding (Figure 4). These MF terms indicated the involvement of these genes in signaling pathways through their roles as ligands, receptors, and cytokines, which suggested that the mechanisms by which Ginsenoside Rg3 could influence heart failure pathophysiology through modulation of these molecular functions.

**Figure 5:**
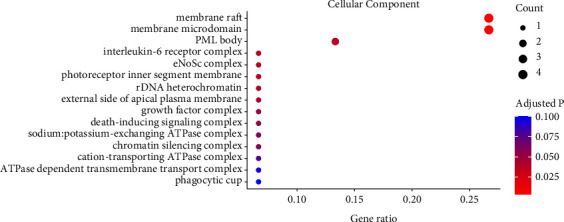
The enriched cellular component terms of the Gene Ontology enrichment.

For the cellular component category, the enriched terms included membrane raft, membrane microdomain, interleukin-6 receptor complex, eNoSc complex, PML body, photoreceptor inner segment membrane, rDNA heterochromatin, external side of apical plasma membrane, and growth factor complex (Figure 5).

### 3.4. Kyoto Encyclopedia of Genes and Genomes Enrichment

**Figure 6:**
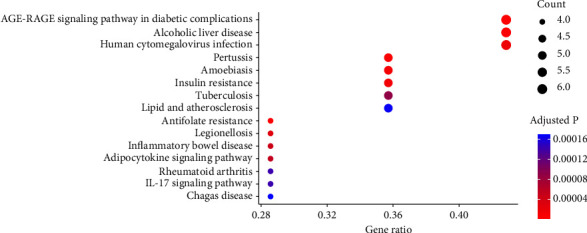
The enriched terms of Kyoto Encyclopedia of Genes and Genomes enrichment.

The KEGG pathway enrichment analysis of the drug-disease interaction genes revealed their significant associated with various pathways implicated in disease processes and metabolic signaling. Specifically, the analysis identified AGE-RAGE signaling pathway in diabetic complications, alcoholic liver disease, pertussis, antifolate resistance, amoebiasis, insulin resistance, human cytomegalovirus infection, legionellosis, inflammatory bowel disease, adipocytokine signaling pathway, tuberculosis, rheumatoid arthritis, IL-17 signaling pathway, and lipid and atherosclerosis (Figure 6). The enriched pathways suggest that Ginsenoside Rg3 may exert cardioprotective effects through multiple mechanisms, such as modulation of metabolic processes, inflammation, and immune responses.

### 3.5. Effects of Ginsenoside Rg3 on the Expression of Hub Genes in Isoproterenol-Treated H9c2 Cells

**Figure 7:**
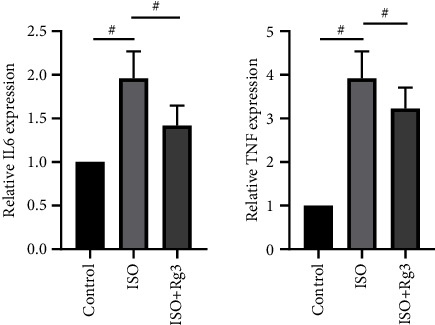
The expression of IL-6 and TNF after the treatment of Ginsenoside Rg3. # refers to statistically significant. IL-6 and TNF were elevated after isoproterenol treatment, while Ginsenoside Rg3 application was lower in their elevation. ^*∗*^: *P* < 0.05.

The mRNA expression levels of IL-6 and TNF were measured by RT-PCR. As shown in Figure 7, the expression of IL-6 and TNF was elevated after isoproterenol treatment, while Ginsenoside Rg3 significantly reduced the levels of IL-6 and TNF.

## 4. Discussion

Ginsenoside Rg3 has garnered wide attention owing to its medicinal properties of anti-inflammation [5], antioxidant [6], and antiageing effects [8]. This study performed network pharmacology to elucidate the protective effect of Ginsenoside Rg3 on heart failure and the underlying mechanisms. Inflammation has been recognized as a contributing role in the development and progression of heart failure [36]. The levels of systemic inflammation are associated with heart failure-related complications and the incidence of poor survival outcomes independent of heart function [36, 37]. Based on network pharmacology analysis and the subsequent enrichment analysis, our results suggested that Ginsenoside Rg3 may be used to treat heart failure via anti-inflammation effects, which potentially involved regulation of interleukin-8 production, regulation of immune effector process, positive regulation of IL-6 production, regulation of interleukin-8 production, positive regulation of immune effector process, cytokine receptor binding, cytokine activity, adipocytokine signaling pathway, and interleukin-17 signaling pathway. Moreover, IL-6 was identified as one of the potential hub genes underlying the antiheart failure effect of Ginsenoside Rg3. IL-6, a classic pleiotropic cytokine, which influences various biological responses in immune system cells and cardiomyocytes following injury [38]. The upregulated expression of IL-6 in isoproterenol-treated H9c2 cells was reversed by Ginsenoside Rg3, which encourages the following investigation with a focus on IL-6.

The cardioprotective anti-inflammation effects of Ginsenoside Rg3 have also been previously explored [15, 16, 39]. A study on cardiomyocyte cell lines AC16 and HCM evaluated the effects of Ginsenoside Rg3 in inhibiting myocardial hypertrophy [16]. Myocardial hypertrophy was induced in rats through transverse aortic constriction and *in vitro* using angiotensin II (Ang II). The administration of Ginsenoside Rg3 revealed a dose-dependent mitigation in Ang II-induced myocardial hypertrophy and fibrosis. In transverse aortic constriction-induced heart failure rats, Rg3 alleviated myocardial hypertrophy and fibrosis by suppressing inflammation and oxidative stress. Significantly, Rg3 was found to inhibit the NLRP3-ASC-Caspase1 inflammasome and reduce oxidative stress in cardiomyocytes. Following mechanistic analyses suggested that the protective effects of Rg3 were mediated through suppressing NF-*κ*B activation and enhancing SIRT1 expression, while the inhibition of SIRT1 significantly reversed the beneficial effects of Rg3 [16]. Overall, Rg3 combats Ang II-induced myocardial hypertrophy by deactivating the NLRP3 inflammasome and oxidative stress via the SIRT1/NF-*κ*B pathway [16].

Consistently, it was reported that Ginsenoside Rg3 improved both cardiac function and calcium homeostasis in mice with heart failure induced by transverse aortic constriction, which was attributed to elevated SUMOylation levels and enhanced SERCA2a activity [15]. Rg3 counteracted mitigated isoproterenol-induced calcium overload in HL-1 cells. The cardioprotective role of Rg3 was compromised in SUMO1 knockout mice, underscoring the critical role of SUMO1 in mediating the effects of Rg3. Additionally, mutations at the SUMOylation sites of SERCA2a abrogated its beneficial impact on isoproterenol-induced calcium dysregulation in HL-1 cells. These findings suggest that Ginsenoside Rg3 exerts a positive influence on the calcium cycling in cardiomyocytes of heart failure mice via SUMO1, which highlighted a potential therapeutic approach for heart failure through targeting SERCA2a SUMOylation [15].

The administration of Ginsenoside Rg3 for cardiovascular disease has yielded promising results. Li et al. [40] developed reactive oxygen species-responsive nanoparticles to encapsulate and deliver Ginsenoside Rg3 efficiently. Through molecular docking and gene silencing techniques, FoxO3a was identified as the critical therapeutic target of Ginsenoside Rg3. In a rat model of ischemia-reperfusion, nanoparticles loaded with Rg3 significantly enhanced cardiac function and diminished infarct size. The cardioprotective effects of Ginsenoside Rg3 were ascribed to its targeting of FoxO3a, leading to the inhibition of oxidative stress, inflammation, and fibrosis. This novel ROS-responsive drug delivery system offers a promising therapeutic approach for ischemic conditions based on Ginsenoside Rg3 [40].

As a preliminary study, our results provide a general overview of the anti-inflammation effects of Ginsenoside Rg3, with a focus on its potential application against heart failure. Multiple inflammation-related pathways were enriched, and the expression levels of IL-6 and TNF were significantly reversed after Ginsenoside Rg3 treatment. Our results indicate that Ginsenoside Rg3 might be a potential treatment against heart failure. Still, it should be noted that our results were primarily acquired by computational analysis based on databases, with limited experimental validation. Also, heart failure can be caused by various reasons, and there is a long to go before we can ensure Ginsenoside Rg3 can benefit which subtype of heart failure or population indeed. Therefore, our results should be taken with caution. Despite the overall anti-inflammation effect indicated by this study, more in-depth investigations on Ginsenoside Rg3 remain to be performed to further reveal its role in inhibiting heart failure.

## 5. Conclusion

This study suggests that Ginsenoside Rg3 exhibits cardioprotective effects against heart failure, potentially owing to its anti-inflammatory properties. Our results indicate that Ginsenoside Rg3 might be a promising treatment against heart failure. However, more in-depth research is necessary to fully elucidate the underlying mechanisms of Ginsenoside Rg3, with a particular focus on its anti-inflammatory effects.

## Figures and Tables

**Table 1:** The gene symbols of these drug-disease interaction genes.

Symbol	Symbol full name
ABCG2	ATP binding cassette subfamily G member 2 (junior blood group)
AR	Androgen receptor
ATP1A1	ATPase Na+/K+ transporting subunit Alpha 1
CASP3	Caspase 3
CCN2	Cellular communication network factor 2
IL1B	Interleukin 1 beta
IL-6	Interleukin 6
LGALS3	Galectin 3
NOS2	Nitric oxide synthase 2
PCK1	Phosphoenolpyruvate carboxykinase 1
PPARGC1A	PPARG coactivator 1 alpha
SIRT1	Sirtuin 1
STAT3	Signal transducer and activator of transcription 3
TNF	Tumor necrosis factor
VEGFA	Vascular endothelial growth factor A

## Data Availability

The datasets analyzed during the current study are available in the PubChem, SwissTargetPrediction, Comparative Toxicogenomics Database, Therapeutic Target Database, DisGeNET, and PharmGKB databases.

## Supplementary Materials

Supplementary Materials Supplement Table 1: Primer sequences for real-time PCR in this study. Supplement Table 2: GeneCards summary of the drug-disease interaction genes.
