# Supplementary material for: The Pharmacological Mechanisms Underlying the Protective Effect of Ginsenoside Rg3 against Heart Failure
Source: Cardiol Res Pract. 2024 Jul 30;2024:3373410. doi: 10.1155/2024/3373410 (PMC11303059; doi:10.1155/2024/3373410)
Supplement: Supplementary Materials — Supplement Table 1: Primer sequences for real-time PCR in this study. Supplement Table 2: GeneCards summary of the drug-disease interaction genes. [file 3373410.f1.zip › Supplement Table 1 (1).docx]

**Supplement Table 1.** Primer sequences for real-time PCR in this study.

| Gene | Forward primer | Reverse primer |
| --- | --- | --- |
| GAPDH | CAAGGTCATCCATGACAACTTTG | GGGCCATCCACAGTCTTCTG |
| IL-6 | ACAAAGCCAGAGTCCTTCAGAG | ACCACAGTGAGGAATGTCCAC |
| TNF-α | TGTGCCTCAGCCTCTTCTCATT | TTGTCACTCGAGTTTTGAGAAGATG |
