# Supplementary material for: The Pharmacological Mechanisms Underlying the Protective Effect of Ginsenoside Rg3 against Heart Failure
Source: Cardiol Res Pract. 2024 Jul 30;2024:3373410. doi: 10.1155/2024/3373410 (PMC11303059; doi:10.1155/2024/3373410)
Supplement: Supplementary Materials — Supplement Table 1: Primer sequences for real-time PCR in this study. Supplement Table 2: GeneCards summary of the drug-disease interaction genes. [file 3373410.f1.zip › Supplement Table 2.docx]

**Supplement Table 2**. GeneCards summary of the drug-disease interaction genes.

| Symbol | GeneCards Summary |
| --- | --- |
| ABCG2 | ABCG2 (ATP Binding Cassette Subfamily G Member 2 (Junior Blood Group)) is a Protein Coding gene. Diseases associated with ABCG2 include Uric Acid Concentration, Serum, Quantitative Trait Locus 1 and Blood Group, Junior System. Among its related pathways are Abacavir transport and metabolism and. Acetaminophen Pathway (therapeutic doses), Pharmacokinetics. Gene Ontology (GO) annotations related to this gene include protein homodimerization activity and ATPase activity. An important paralog of this gene isABCG1. |
| AR | AR (and rogen Receptor) is a Protein Coding gene. Diseases associated with AR include and rogen Insensitivity, Partial and Spinal and Bulbar Muscular Atrophy, X-Linked 1. Among its related pathways are Activated PKN1 stimulates transcription of AR (and rogen receptor) regulated genes KLK2 and KLK3 and Akt Signaling. Gene Ontology (GO) annotations related to this gene include DNA-binding transcription factor activity and chromatin binding. An important paralog of this gene isNR3C2. |
| ATP1A1 | ATP1A1 (ATPase Na+/K+ Transporting Subunit Alpha 1) is a Protein Coding gene. Diseases associated with ATP1A1 include Charcot-Marie-Tooth Disease, Axonal, Type 2Dd and Hypomagnesemia, Seizures, and Mental Retardation 2. Among its related pathways are Aldosterone synthesis and secretion and Aldosterone-regulated sodium reabsorption. Gene Ontology (GO) annotations related to this gene include nucleotide binding and protein domain specific binding. An important paralog of this gene isATP1A3. |
| CASP3 | CASP3 (Caspase 3) is a Protein Coding gene. Diseases associated with CASP3 include Oropharynx Cancer and Helicobacter Pylori Infection. Among its related pathways are 14-3-3 and Regulation of BAD Activity and 4-1BB Pathway. Gene Ontology (GO) annotations related to this gene include peptidase activity and cysteine-type peptidase activity. An important paralog of this gene isCASP7. |
| IL1B | IL1B (Interleukin 1 Beta) is a Protein Coding gene. Diseases associated with IL1B include Gastric Cancer, Hereditary Diffuse and Toxic Shock Syndrome. Among its related pathways are MAPK Pathway and Tuberculosis. Gene Ontology (GO) annotations related to this gene include protein domain specific binding and interleukin-1 receptor binding. An important paralog of this gene isIL36RN. |
| IL6 | IL6 (Interleukin 6) is a Protein Coding gene. Diseases associated with IL6 include Kaposi Sarcoma and Rheumatoid Arthritis, Systemic Juvenile. Among its related pathways are Tuberculosis and ErbB signaling pathway. Gene Ontology (GO) annotations related to this gene include signaling receptor binding and growth factor activity. |
| LGALS3 | LGALS3 (Galectin 3) is a Protein Coding gene. Diseases associated with LGALS3 include Follicular Adenoma and Papillary Carcinoma. Among its related pathways are Innate Immune System and Hedgehog signaling events mediated by Gli proteins. Gene Ontology (GO) annotations related to this gene include chemoattractant activity. An important paralog of this gene isLGALS9. |
| NOS2 | NOS2 (Nitric Oxide Synthase 2) is a Protein Coding gene. Diseases associated with NOS2 include Malaria and Meningioma, Familial. Among its related pathways are Tuberculosis and Effects of Nitric Oxide. Gene Ontology (GO) annotations related to this gene include protein homodimerization activity and oxidoreductase activity. An important paralog of this gene isNOS1. |
| PCK1 | PCK1 (Phosphoenolpyruvate Carboxykinase 1) is a Protein Coding gene. Diseases associated with PCK1 include Phosphoenolpyruvate Carboxykinase Deficiency, Cytosolic and Pepck 1 Deficiency. Among its related pathways are Glucose / Energy Metabolism and Developmental Biology. Gene Ontology (GO) annotations related to this gene include GTP binding and GDP binding. An important paralog of this gene isPCK2. |
| PPARGC1A | PPARGC1A (PPARG Coactivator 1 Alpha) is a Protein Coding gene. Diseases associated with PPARGC1A include Amyotrophic Lateral Sclerosis 1 and Huntington Disease. Among its related pathways are Glucose / Energy Metabolism and Developmental Biology. Gene Ontology (GO) annotations related to this gene include nucleic acid binding and RNA binding. An important paralog of this gene isPPARGC1B. |
| SIRT1 | SIRT1 (Sirtuin 1) is a Protein Coding gene. Diseases associated with SIRT1 include Aging and Periapical Periodontitis. Among its related pathways are Glucose / Energy Metabolism and HIF-2-alpha transcription factor network. Gene Ontology (GO) annotations related to this gene include identical protein binding and transcription factor binding. An important paralog of this gene isSIRT3. |
| STAT3 | STAT3 (Signal Transducer and Activator Of Transcription 3) is a Protein Coding gene. Diseases associated with STAT3 include Hyper-Ige Recurrent Infection Syndrome 1, Autosomal Dominant and Autoimmune Disease, Multisystem, Infantile-Onset, 1. Among its related pathways are MAPK Pathway and ErbB signaling pathway. Gene Ontology (GO) annotations related to this gene include DNA-binding transcription factor activity and sequence-specific DNA binding. An important paralog of this gene isSTAT1. |
| TNF | TNF (Tumor Necrosis Factor) is a Protein Coding gene. Diseases associated with TNF include Asthma and Malaria. Among its related pathways are Tuberculosis and Developmental Biology. Gene Ontology (GO) annotations related to this gene include identical protein binding and cytokine activity. An important paralog of this gene isLTA. |
| VEGFA | VEGFA (Vascular Endothelial Growth Factor A) is a Protein Coding gene. Diseases associated with VEGFA include Microvascular Complications Of Diabetes 1 and Poems Syndrome. Among its related pathways are ErbB signaling pathway and G-protein signaling Ras family GTPases in kinase cascades (scheme). Gene Ontology (GO) annotations related to this gene include protein homodimerization activity and protein heterodimerization activity. An important paralog of this gene isPGF. |
